# Concentrated Colloidal Dispersion of Nickelladithiolene Coordination Nanosheet Realized by an Alkylated Modulator

**DOI:** 10.3390/nano16030191

**Published:** 2026-01-30

**Authors:** Naoya Fukui, Yu Endo, Miyu Ito, Kenji Takada, Hiroaki Maeda, Hiroshi Nishihara

**Affiliations:** 1 Research Institute for Science and Technology, Tokyo University of Science, 2641 Yamazaki, Noda 278-8510, Japan; takada.k.ag@rs.tus.ac.jp (K.T.); h-maeda@rs.tus.ac.jp (H.M.); 2 Department of Pure and Applied Chemistry, Faculty of Science and Technology, Tokyo University of Science, 2641 Yamazaki, Noda 278-8510, Japan

**Keywords:** coordination nanosheet, nickelladithiolene, colloid, conductive ink

## Abstract

Nickelladithiolene nanosheet, Ni_3_BHT, is a two-dimensional material composed of nickel ions and benzenehexathiol (BHT). Ni_3_BHT has attracted considerable attention owing to its electrical conductivity. Although conventional Ni_3_BHT is obtained as a solid film or powder, recent studies have explored methods for handling Ni_3_BHT as a liquid ink, which facilitates industrial applications. One such method involves adding a modulator ligand to control the morphology of Ni_3_BHT. In this study, we developed a novel modulator ligand, 4,5-dihexylbenzene-1,2-dithiol (**CL1**), which afforded a more stable and concentrated Ni_3_BHT dispersion than those previously reported. Further investigations suggest that **CL1** is incorporated not only at the termini but also within the interior of the Ni_3_BHT nanoflakes, based on the consistent interpretation of spectroscopic and morphological data, in the dispersion via the addition of an adequate amount of a modulator. The application of the Ni_3_BHT dispersion as a conductive ink was demonstrated. The Ni_3_BHT ink exhibited the highest electrical conductivity and colloidal stability at a **CL1**/BHT ratio of 0.3. These findings pave the way for potential applications of Ni_3_BHT in various industries.

## 1. Introduction

Metal–organic frameworks (MOFs) are composed of metal ions coordinated by organic molecules, which serve as linkers [1,2,3,4,5,6,7,8,9]. They have attracted considerable attention because of their interesting properties and high structural and functional designability. Among them, two-dimensional frameworks in which planar ligand molecules are linked by planar complex moieties are called coordination nanosheets (CONASHs) [10,11,12,13,14,15,16,17,18,19,20]. The presence of delocalized π–d conjugated electronic states endows CONASHs with high electrical conductivity, placing it among the representative conductive metal–organic frameworks [12]. Significant efforts have been made towards its application in electronic devices [15], electrochemical catalysis [21,22,23,24], energy storage [25,26], and sensors [27,28,29,30].

CONASHs usually take the form of continuous films synthesized at a liquid–liquid interface or a powder obtained by precipitation. In these macroscopic forms, their properties reflect bulk, macroscopic characteristics. However, nanoparticles of inorganic materials, such as quantum dots, exhibit remarkable properties different from those of macroscopic materials [31,32]. Furthermore, nanoparticles can be dispersed in a solvent, facilitating easy handling. The fascinating properties of such inorganic nanoparticles have motivated us to synthesize nanoscale CONASHs (nanoflakes), owing to their potential chemical and physical properties. Our recent study revealed that nanoparticles of Ni_3_BHT can be obtained using benzene-1,2-dithiol as an additive that suppresses the further polymerization of Ni_3_BHT [33,34]. These Ni_3_BHT nanoparticles served as more efficient co-catalyst for water splitting than μm-sized Ni_3_BHT sheets. However, studies on CONASHs are still in their infancy, and further research is required.

In this study, we synthesized Ni_3_BHT nanoparticles modified with 4,5-dihexylbenzene-1,2-dithiol, **CL1**, as a capping ligand. The obtained nanoparticles were terminated with alkyl chains from the capping ligand, thereby improving the stability of the nanoparticles dispersed in the organic solvent. Various measurements indicate that **CL1** plays dual roles in the formation of Ni_3_BHT nanoparticles: partial substitution of BHT ligands within the coordination network and modification of the flake termini, stabilizing the dispersion when an adequate amount of **CL1** is added to the reaction system. In contrast, when the amount of **CL1** was insufficient, alkyl modification of the Ni_3_BHT nanoparticles was imperfect, leading to aggregation. The Ni_3_BHT dispersion serves as a conductive ink, facilitating future industrial applications.

## 2. Materials and Methods

**Materials** 

All chemicals were purchased from Tokyo Chemical Industry Co., Ltd. (Tokyo, Japan), Kanto Chemical Co. (Tokyo, Japan), Sigma-Aldrich Co. LLC (St. Louis, MO, USA), Wako Pure Chemical Industries Ltd. (Osaka, Japan), and Taiyo Nippon Sanso Corp. (Tokyo, Japan), unless otherwise stated. All samples were used without further purification. Water was purified by using a Milli-Q purification system (Merck KGaA, Darmstadt, Germany). 1,2-dibromo-4,5-dihexylbenzene [35] and benzenehexathiol [36] were synthesized according to previously reported methods.

**Instruments** 

Transmission electron microscopy (TEM) images were collected at 100 kV using an H-7650 microscope (Hitachi, Tokyo, Japan). TEM samples were prepared by drop-casting a sample dispersion diluted 50 times on a copper grid with supporting carbon films (Nisshin EM, Tokyo, Japan). Dynamic light scattering (DLS) was performed at a scattering angle of 173° at 25 °C and analyzed under the assumption of a broad dispersion using SZ-100 (HORIBA, Kyoto, Japan) after diluting the sample dispersion 50 times with a mixed solvent (tetrahydrofuran (THF): MeOH = 1:1 (*v*/*v*)). The viscosity of the mixed solvent was estimated by interpolating values from literature [37]. Powder X-ray diffraction (PXRD) measurements were performed at BL44B2 of SPring-8 at a wavelength of 0.8 Å. PXRD patterns were reproduced using VESTA software (Ver. 3.5.8) [38]. X-ray photoelectron spectroscopy (XPS) measurements were performed using a PHI 5000 VersaProbe and VersaProbe III (ULVAC-PHI, Kanagawa, Japan). Al Kα (15 kV, 25 W) was used as the X-ray source, and the beam was focused on an area of 100 μm^2^. The XP spectra were analyzed using MultiPak Software (Ver. 9.9.3) and standardized using a C 1s peak at 284.6 eV. Infrared absorption (IR) spectra were collected using an FT/IR-6100 spectrophotometer (JASCO, Tokyo, Japan) and attenuated total reflection (ATR) method. The background signal was subtracted using the software. Raman spectra were collected using an NRS-5500 (JASCO, Tokyo, Japan) instrument with a 532 nm excitation laser. Ultraviolet-visible-near-infrared absorption (UV-Vis-NIR) spectra were collected using a V-700 (JASCO, Tokyo, Japan) instrument in a 10 mm quartz glass cell with an integrating sphere. Centrifugation was performed using a CS150GXII centrifuge (Eppendorf Himac Technologies Co., Ltd., Ibaraki, Japan). Electrical conductivity measurements were performed under Ar using a custom-ordered probe system within a glove box (Oyama, Hyogo, Japan) connected to a Keithley 2450 SourceMeter (Beaverton, OR, USA) in the two-probe mode. Samples for the electrical conductivity measurements were prepared by drop-casting the sample dispersion onto an interdigitated gold electrode (GEOMATEC, Kanagawa, Japan). Atomic force microscopy (AFM) images were collected using an Agilent Technologies 5500 Scanning Probe Microscope (Santa Clara, CA, USA) and processed using Gwyddion 2.69 [39].

**Synthesis of 1,2-bis(methylthio)-4,5-dihexylbenzene (1)** 

1,2-Dibromo-4,5-dihexylbenzene (3.342 g, 9.87 mmol) and excess sodium methanethiolate (6.2 g, 88 mmol) were added to 50 mL of dry 1,3-dimethyl-2-imidazolidinone. The mixture was stirred and refluxed overnight in a nitrogen atmosphere. This resulted in a brownish-yellow suspension. After cooling to room temperature, 6 mL iodomethane was added dropwise. Hexane and ethyl acetate (*v*/*v* = ~ 4:1) were added to the reaction mixture, and the mixture was repeatedly washed with water, followed by evaporation. The crude material was purified using silica gel column chromatography (hexane: ethyl acetate = 8:1), and the second band was collected. A colorless oil was obtained (649 mg, 23%). ^1^H-NMR (400 MHz, CDCl_3_, ppm): *δ* = 0.90 (t, *J* = 6.78 Hz, 6H), 1.27–1.42 (m, 12H), 1.50–1.59 (m, 4H), 2.45 (s, 6H), 2.56 (t, *J* = 7.98 Hz, 4H), 7.01 (s, 2H). ^13^C-NMR (100 MHz, CDCl_3_, ppm): *δ* = 14.23, 16.83, 22.76, 29.52, 31.44, 31.85, 32.51, 128.63, 134.40, 139.08.

**Synthesis of 4,5-dihexylbenzene-1,2-dithiol (CL1)** 

All processes except for collection were performed using the Schlenk technique. An excess amount of sodium (0.5 g, 22 mmol) was dissolved in liquid ammonia (~100 mL) and cooled in a dry ice/acetone bath. The ammonia solution turned dark blue. **1** (301.35 mg, 0.972 mmol) dissolved in degassed THF (4 mL) was added dropwise to liquid ammonia and stirred for 4 h. Degassed methanol (2 mL) was carefully added to liquid ammonia to quench the excess sodium. When the reaction mixture was allowed to warm to room temperature, the blue color disappeared. The product was diluted with 90 mL of degassed water and washed repeatedly with degassed diethyl ether. Ten milliliters of degassed HCl aq. (35 wt%) was added, and the solution was extracted with diethyl ether under an ambient atmosphere. The organic phase was dried with Na_2_SO_4_ and evaporated to obtain a greenish-yellow oil (200 mg, 72%). ^1^H-NMR (400 MHz, CDCl_3_, ppm): *δ* = 0.89 (t, *J* = 7.03 Hz, 6H), 1.25–1.39 (m, 12H), 1.47–1.55 (m, 4H), 2.49 (t, *J* = 8.01 Hz, 4H), 3.64 (s, 2H), 7.15 (s, 2H). ^13^C-NMR (100 MHz, CDCl_3_, ppm): *δ* =14.25, 22.75, 29.48, 31.27, 31.84, 32.24, 127.81, 132.08, 140.09.

**Synthesis of Ni_3_BHT-x** 

**Ni_3_BHT-x** was synthesized by adding 10 mL of a 4.5 mM Ni(OAc)_2_ methanol solution to 10 mL of a stirred solution containing 1.5 mM benzenehexathiol (BHT) and 1.5 × x mM **CL1** THF under an inert atmosphere at room temperature. The reaction proceeded immediately, yielding a dark brown dispersion, with or without black precipitation. For all measurements except UV–Vis–NIR spectroscopy, the resulting **Ni_3_BHT-x** were collected by evaporating the solvent to promote aggregation, followed by filtration through a polytetrafluoroethylene (PTFE) membrane with a pore size of 450 nm. The collected black solids (**Ni_3_BHT-x**) were thoroughly washed with THF and methanol and dried in vacuo.

**Synthesis of Ni_3_BHT-BDT-x** 

**Ni_3_BHT-BDT-x** was synthesized by the same method as **Ni_3_BHT-x,** by using benzene-1,2-dithiol instead of **CL1**.

**Preparation of the sample for UV-Vis-NIR spectroscopy** 

“As-prepared” samples were obtained by diluting the freshly **Ni_3_BHT-x** dispersion 50 times with a mixed solvent of THF and MeOH (1:1 (*v*/*v*)) without purification. 5 mL of the as-prepared **Ni_3_BHT-x** was centrifugated for 3 h at 110 krpm. The supernatant was diluted 50 times with the mixed solvent for measurements. The black precipitate was washed with THF and methanol, sonicated to redisperse **Ni_3_BHT-x** in 5 mL of the mixed solvent, and diluted 50 times with the mixed solvent for measurement.

## 3. Results

**Figure 1:**
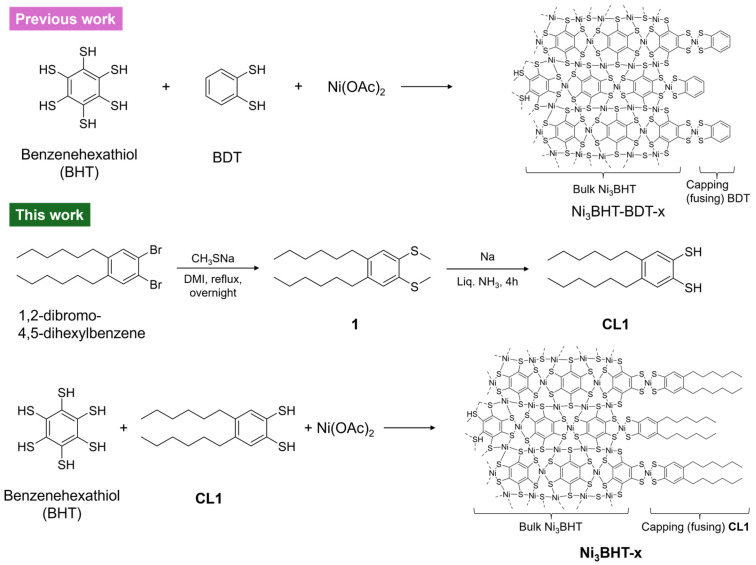
Schematic illustration of previous work (**Ni_3_BHT-BDT-x**) and this work (the novel modulator **CL1** and the colloid **Ni**_3_**BHT-x**).

Modifying the termini of nanostructures with alkyl chains is a common strategy to stabilize the nanostructures dispersed in solvents [40]. In this study, we synthesized a novel capping ligand, 4,5-dihexylbenzene-1,2-dithiol (**CL1**) and applied it to Ni_3_BHT (Figure 1). **CL1** was obtained from 1,2-dibromo-4,5-dihexylbenzene via the introduction of SCH_3_ by nucleophilic substitution to synthesize 1,2-dimethylthio-4,5-dihexylbenzene (**1**), followed by the deprotection of the methyl groups by Birch-type reduction. Among the protecting groups examined, methyl groups were the best, while benzyl groups only replaced one bromine in 1,2-dibromo-4,5-dihexylbenzene.

**Figure 2:**
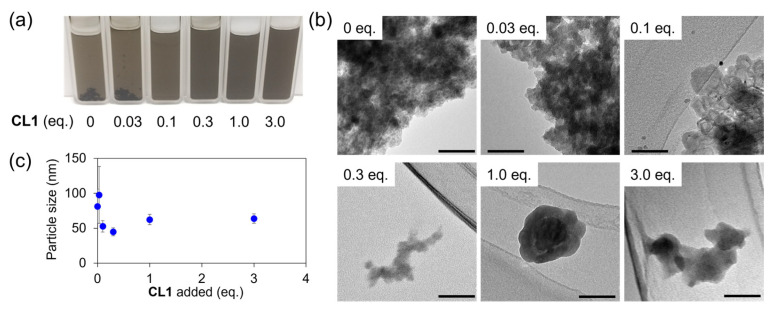
(**a**) Colloidal dispersion of **Ni_3_BHT-x** (*x* = 0, 0.03, 0.1, 0.3, 1.0, and 3.0) in quartz cells. (**b**) TEM images of **Ni_3_BHT-x**. The scale bar is 50 nm. (**c**) Particle size of **Ni_3_BHT-x** as measured by DLS.

To investigate the effect of **CL1** on the formation of Ni_3_BHT colloids, we prepared dispersions of Ni_3_BHT nanoflakes (**Ni_3_BHT-x**) by mixing a BHT/THF solution with *x* equivalents of **CL1** (*x* = 0, 0.03, 0.1, 0.3, 1.0, and 3.0) relative to BHT in the solution and a methanol solution of 3 equivalents of nickel acetate. Figure 2a shows the resulting **Ni_3_BHT-x** dispersions. All dispersions were greenish-brown, similar to Ni_3_BHT colloids obtained in a previous study [41]. The color of **Ni_3_BHT-x** was darker than that of the Ni_3_BHT dispersion prepared in a previous study without a modulator, because the concentration of Ni_3_BHT was ten times as large as that in the previous study. In samples with low **CL1** contents (*x* = 0, 0.03), the product aggregated and formed a black gel-like precipitate. Although the Ni_3_BHT colloids were stable in dilute solutions, as the concentration increased, the attractive interactions between the flakes became stronger, and the colloidal state could no longer be maintained. In the samples with a sufficient **CL1** content (*x* ≥ 0.1), no visible solid precipitations were observed immediately after synthesis. After one month, aggregation occurred eventually at *x* = 0.1, but the samples with *x* ≥ 0.3 maintained a uniform colloid state, which lasted for up to three months (Figure S1). These results demonstrate that **CL1** works as a modulator that solubilizes Ni_3_BHT in a methanol/THF system, making it possible to prepare colloids containing Ni_3_BHT at concentrations 10 times higher than those in conventional Ni_3_BHT colloids. Benzene-1,2-dithiol (BDT) has also been reported to solubilize Ni_3_BHT in ethanol [34]. However, under the same synthesis conditions as those used in this study, aggregation occurred one month after synthesis at all additive amounts (Figure S1). Therefore, **CL1** is a superior modulator compared to BDT in terms of stability. Figure 2b shows the TEM images of the **Ni_3_BHT-x** dispersion (supernatant, if a precipitate is present). At low **CL1** concentrations (*x* ≤ 0.1), the dispersion aggregated to form clumps, whereas at high **CL1** concentrations (*x* ≥ 0.3), flakes of approximately 100 nm were observed. It is reasonable that under low **CL1** concentrations, where precipitation occurs, the flake size is large, whereas under conditions where precipitates are not formed, the flake size is small. The tendency of the flake size to decrease with increasing **CL1** addition is also evident in dynamic light scattering (DLS) measurements (Figure 2c).

DLS measurements qualitatively capture the trend of decreasing flake size with increasing **CL1** content. However, the flake size measured by DLS did not match that by TEM images, particularly at *x* = 0 and 0.03, where larger flakes were observed by TEM, owing to the nonspherical, sheet-like morphology of Ni_3_BHT nanoflakes, which violates the spherical particle assumption inherent to DLS analysis. This phenomenon was due to the nonspherical particle shape, which is often observed when measuring two-dimensional materials [42,43]. These results suggest that the addition of **CL1** suppresses the aggregation of Ni_3_BHT flakes. The particle size reduction effect appears to be dominant in the combination of Ni_3_BHT and **CL1;** however, modulators do not always decrease the size of the MOF particles [44,45,46].

**Figure 3:**
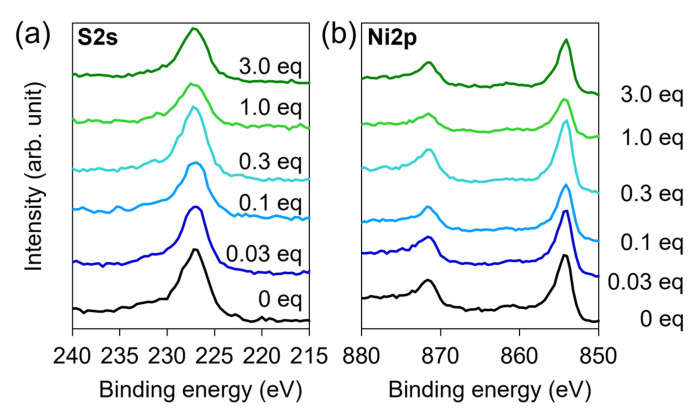
XP spectra of **Ni_3_BHT-x** in (**a**) S2s region and (**b**) Ni2p region.

Figure S2 shows powder X-ray diffraction patterns of **Ni_3_BHT-x**. As is common with nanoparticles, the pattern is broad owing to low crystallinity. To clarify the chemical bonding state, we performed XPS measurements of **Ni_3_BHT-x** (Figure 3a,b and Figure S3). S2s and Ni2p peaks appeared at 227.0 and 854.2 eV, respectively, regardless of the amount of **CL1** added. The XPS of **Ni_3_BHT-0** was consistent with that for Ni_3_BHT synthesized by the liquid–liquid two-phase interface or single-phase synthesis method [16,41]. This suggests that **CL1**, as well as BHT, bonds with Ni and forms a part of a planar network with nickel ions and BHT. The S/Ni ratio estimated from the S2s and Ni2p peak areas varied between 2 and 2.5 depending on the amount of CL1 added, consistent with the ideal S/Ni ratio of 2 calculated from the Ni_3_BHT composition (Figure S3). A more detailed analysis of the S2s peak revealed that it can be divided into two peaks: a main peak at 227.0 eV and peak around 230 eV. The peak at 230 eV is a shake-up peak often observed in metal dithiolene-based materials [14,47,48], and it is derived from highly oxidized sulfur, such as sulfate or disulfide. The peak near 230 eV was present in **Ni_3_BHT-0** but became less prominent with increasing **CL1** content (Figure S4). The area ratio to the main peak decreased with increasing *x* up to *x* = 0.3, then leveled off thereafter (Figure S5). While **Ni_3_BHT-0** without a modulator contained a large amount of highly oxidized S, the progressive addition of **CL1** suppresses oxidized sulfur species at the flake edges, resulting in a relative increase in the sulfur content involved in complex formation, consistent with replacement of oxidation-prone BHT-derived termini by alkylated **CL1** ligands.

**Figure 4:**
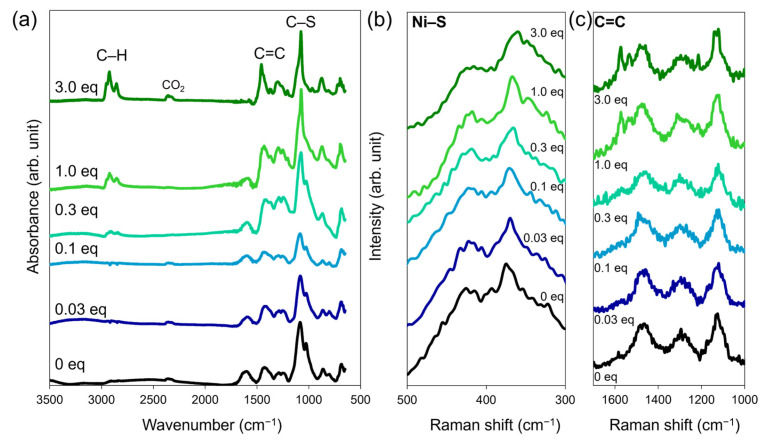
(**a**) IR spectra of **Ni_3_BHT-x**. (**b**,**c**) Raman spectra of **Ni_3_BHT-x** (**b**) from 300 to 500 cm^−1^ and (**c**) from 1000 to 1700 cm^−1^.

We investigated the changes in the vibrational spectra of Ni_3_BHT upon the addition of **CL1** using infrared (IR) absorption and Raman spectroscopy (Figure 4 and Figure S6). The Raman spectrum of the sample without **CL1** (**Ni_3_BHT-0**) was consistent with that of Ni_3_BHT synthesized by a two-phase interfacial reaction or single-phase synthesis, indicating that **Ni_3_BHT-0** is a colloid composed of Ni_3_BHT coordination nanosheets [16]. Neither IR nor Raman spectra showed a peak near 2500 cm^−1^ due to S–H stretching vibrations, suggesting that most of the sulfur from BHT and **CL1** coordinated to nickel ions (Figure 4a and Figure S6). The IR spectra of **Ni_3_BHT-x** (*x* ≥ 0.3) showed an absorption peak near 2900 cm^−1^ (Figure 4a), which was attributed to the C-H stretching of the hexyl or benzene ring of **CL1**. The peak intensity increased with increasing *x*, indicating that the more **CL1** added, the more **CL1** was incorporated into **Ni_3_BHT-x**. In Figure 2b,c, there are no significant changes in the size or shape of **Ni_3_BHT-x** flakes in the *x* ≥ 0.3 region. If **CL1** coordinates only to the termini of the flakes, the amount of **CL1** within the flakes should not change significantly. However, the IR spectra revealed the opposite. Therefore, combining the TEM images and IR spectra of **Ni_3_BHT-x**, it is likely that **CL1** exists not only at the termini of Ni_3_BHT but also in bulk Ni_3_BHT by replacing BHT, and the amount of the latter type of **CL1** increases with increasing *x*. Furthermore, with increasing **CL1,** the C–S and C=C stretching peaks became sharper. In the Raman spectra, the Ni-S stretching peak shifted gradually from 374 cm^−1^ at *x* = 0 to 358 cm^−1^ at *x* = 3.0 (Figure 4b). The absence of peaks around 500 cm^−1^ suggests insignificant S–S bond formation. Furthermore, a new peak, attributable to the stretching vibration of the aromatic ring of **CL1,** appeared at approximately 1570 cm^−1^. These results confirm the fusion of **CL1** with the Ni_3_BHT network of **Ni_3_BHT-x**.

**Figure 5:**
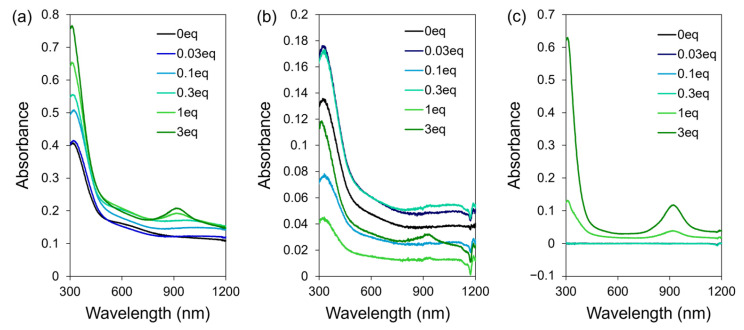
UV-Vis-NIR spectra of (**a**) as-prepared **Ni_3_BHT-x**, (**b**) redispersed **Ni_3_BHT-x**, and (**c**) supernatant of **Ni_3_BHT-x**.

We measured the UV-visible absorption spectrum of **Ni_3_BHT-x** dispersion to gain further insights into its electronic state. Figure 5a shows the UV–vis–NIR spectra of the diluted as-prepared **Ni_3_BHT-x** colloids without further purification. Samples with low **CL1** contents (*x* = 0, 0.03, and 0.1) exhibited significantly broad absorption peaks at approximately 1050 nm. These peaks were attributed to the absorption of Ni_3_BHT [41]. In contrast, samples prepared with higher **CL1** contents (*x* ≥ 1.0) exhibited an additional absorption band at approximately 927 nm. Absorption in the NIR region is characteristic of nickel dithiolene complexes [49,50] and is therefore associated with the formation of dithiolene-based nickel species involving **CL1**. Such species can, in principle, exist in two forms: (i) nickel centers coordinated by both **CL1** and BHT ligands and incorporated into the Ni_3_BHT coordination network, and (ii) discrete molecular species in which two **CL1** ligands coordinate to a nickel center and dissolve in the solvent as a most probably mononuclear Ni–dithiolene complex. To clarify the origin of the absorption band at approximately 927 nm, **Ni_3_BHT-x** dispersions were subjected to centrifugation, followed by washing and attempted redispersion by sonication; however, redispersion was incomplete. The UV-Vis-NIR spectra of the redispersed precipitates are shown in Figure 5b. These spectra retained essentially the same spectral features as those of the original dispersions, dominated by the absorption characteristic of the Ni_3_BHT network, while the peak at 927 nm became relatively less prominent. In contrast, the UV–Vis–NIR spectra of the supernatants collected after centrifugation revealed a clear difference depending on the **CL1** content (Figure 5c). For samples with x ≤ 0.3, no absorption was observed in the 300–1200 nm range. However, for samples with x = 1.0 and 3.0, ultraviolet absorption attributable to free **CL1**, together with a distinct absorption band at approximately 927 nm, was observed. This absorption is reasonably attributed to discrete Ni–dithiolene species present in solution and distinct from the extended Ni_3_BHT coordination network. The absorbance at 927 nm increased proportionally with the amount of **CL1**, reaching 0.038 at *x* = 1.0 and 0.116 at *x* = 3.0. Based on these observations, we infer that when a relatively small amount of **CL1** is added (*x* ≤ 0.3), **CL1** is fully consumed as a component of the Ni_3_BHT-x network, and no discrete molecular species remain in solution. In contrast, when a larger excess of **CL1** is introduced (*x* = 1.0, 3.0), a fraction of the added **CL1** is not incorporated into the Ni_3_BHT network but instead forms discrete Ni–dithiolene species or remains uncoordinated in solution. The absence of the 927 nm absorption band for *x* = 0.3 indicates that, under these conditions, essentially all **CL1** molecules are consumed in the formation of **Ni_3_BHT-0.3**. These results further suggest that coordination of **CL1** and BHT to Ni^2+^ during nanosheet growth is favored over the formation of discrete **CL1**-only complexes at low **CL1** concentrations. At higher **CL1** loadings, however, available BHT-derived thiolate sites at the termini of Ni_3_BHT flakes become saturated, promoting the formation of discrete Ni–dithiolene species in solution.

This interpretation is consistent with the XPS results, which show that increasing **CL1** content progressively suppresses oxidized sulfur species at the flake edges. At lower **CL1** concentrations (*x* < 0.3), increasing **CL1** addition leads to more extensive termination of Ni_3_BHT flakes by **CL1**, concomitantly reducing the amount of oxidation-prone BHT at the edges. At higher **CL1** concentrations (*x* ≥ 0.3), the chemical states at the flake termini no longer change significantly, resulting in a constant ratio of oxidized to coordinated sulfur species.

**Figure 6:**
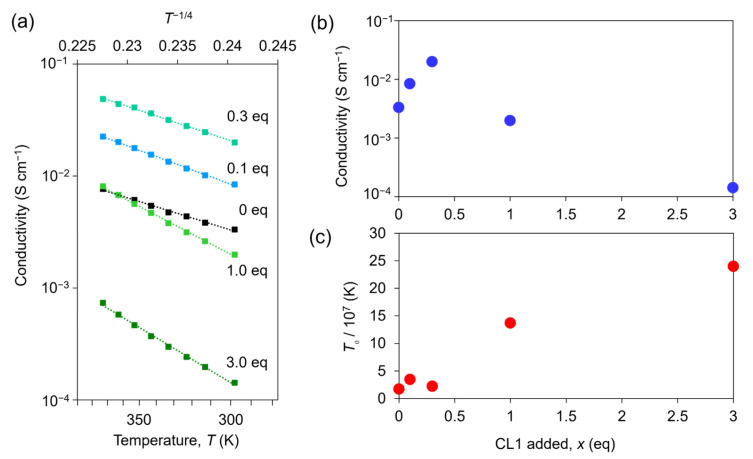
(**a**) Temperature dependence of the electrical conductivity of **Ni_3_BHT-x**. (**b**) Room temperature conductivity of **Ni_3_BHT-x**. (**c**) Fitting parameter (*T*_0_) of 3DVRH for **Ni_3_BHT-x**.

Finally, to investigate its performance as a conductive ink, **Ni_3_BHT-x** was coated onto an interdigitated gold electrode, and its electrical conductivity was measured using the two-terminal method. The coated **Ni_3_BHT-x** formed a film of approximately 100 μm in size with a thickness of approximately 1 μm (Figures S7 and S8). Figure S9 shows the representative current-voltage curve of **Ni_3_BHT-0.3** at room temperature. The linear curve indicates the formation of an ohmic contact between gold and **Ni_3_BHT-0.3**. Finite electrical conductivities were observed for all *x* values, demonstrating that the **Ni_3_BHT-x** dispersion functioned as a conductive ink. The electrical conductivities of all **Ni_3_BHT-x** samples increased with increasing temperature (Figure 6a). This behavior was consistently observed in both Ni_3_BHT nanoflakes and films. Because the logarithm of electrical conductivity is a linear function of the inverse fourth root of temperature, it can be explained by strong localization due to disorder [16,34,41,51]. Hence, we compared the room-temperature electrical conductivities for different values of *x*. At *x* = 0, the electrical conductivity was 3.3 × 10^−3^ S cm^−1^. With increasing **CL1**, the electrical conductivity increased, reaching a maximum of 2.0 × 10^−2^ S cm^−1^ at *x* = 0.3, after which it began to decrease (Figure 6b). The increase in electrical conductivity with increasing **CL1** at smaller *x* values up to 0.3 can be interpreted as a change in the electronic state owing to the substitution of **CL1** for BHT. The substitution of **CL1** injects positive holes because **CL1** has two anionic S atoms while BHT has six anionic S atoms. The injection of holes increases the electrical conductivity of the interior of nanoflakes since Ni_3_BHT is a p-type material [16]. A phenomenon similar to that is reported for Ni_3_BHT substituted with BDT [33]. Note that **CL1** can also work as a scattering center by partially breaking the Ni_3_BHT network and consequently degrade the carrier mobility. In this smaller **CL1** concentration regime, the effect of increasing hole doping overwhelms that of increasing scattering centers. However, when *x* > 0.3, excessive addition of **CL1** led to decrease in the electrical conductivity. In this regime, the substituted **CL1** works as random disorders in the Ni_3_BHT network rather than as dopants, resulting in a random potential much stronger than inherent one in **Ni_3_BHT-0**. The carriers are forced to be trapped in the random potential, which leads to strong localization. This interpretation is consistent with the experimentally observed increase in the *T*_0_ parameter and does not require assumptions beyond the structural disorder introduced by excessive ligand substitution. The decrease in the electrical conductivity by excessive doping is generally found also in other materials [52,53]. The degree of localization can be evaluated using the parameter *T*_0_, which characterizes localization when the temperature dependence of the electrical conductivity is expressed as σ = σ_0_ exp(−(*T*_0_/*T*)^1/4^), assuming a three-dimensional variable range hopping (3DVRH) conduction mechanism [54]. Above *x* = 0.3, *T*_0_ increases rapidly, suggesting stronger carrier localization and more severe structural disorder than in the samples with *x* below 0.3 (Figure 6c).

## 4. Discussion

**Figure 7:**
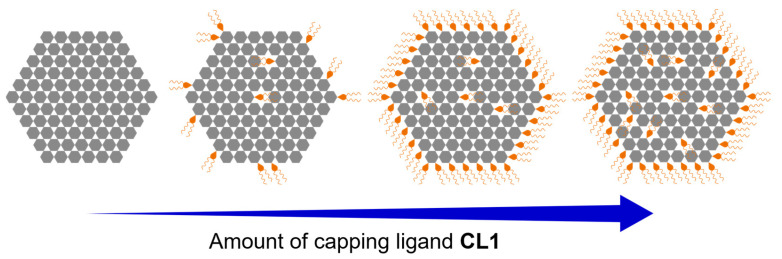
Schematic illustration of the estimated structure of **Ni_3_BHT-x**. The gray hexagon and orange droplets with wavy lines indicate BHT and **CL1** molecules, respectively.

The formation of Ni_3_BHT colloids with **CL1** can be rationalized by the following conceptual model (Figure 7). In the absence of **CL1**, Ni_3_BHT micronuclei consisting of only BHT and Ni ions were formed. These terminals, including thiol groups, are highly active, and their concentration is 10 times higher than that of stable Ni_3_BHT colloids. Therefore, they cannot be stably dispersed in the solvent and can only undergo aggregation and precipitation. When a small amount of **CL1** is present in the reaction system, complexation by BHT ligands with Ni^2+^ mainly proceeds with occasional complexation by BHT and **CL1** with Ni^2+^, resulting in the formation of Ni_3_BHT nanoflakes in which some of BHT is replaced by **CL1**. XPS results suggested that the BHT termini were present when the concentration of **CL1** was low. Therefore, Ni_3_BHT flakes grow with **CL1** partially replacing BHT until BHT is consumed to some extent, followed by the edge modification of **CL1** which was not incorporated into the Ni_3_BHT flakes. If the degree of modification is low because of insufficient **CL1**, the nuclei aggregate and eventually precipitate (*x* = 0.03 and 0.1). If fully modified, owing to sufficient **CL1**, they are stably dispersed (*x* = 0.3). When excess **CL1** is present in the system, Ni_3_BHT flakes are produced, and more BHT is replaced by **CL1**. All the termini get modified with **CL1**, and the remaining **CL1** forms complexes (*x* = 1.0 and 3.0). In this study, the optimal composition for producing Ni_3_BHT colloids with high colloidal stability, high concentration, and minimal byproducts was achieved using a molar ratio of BHT:Ni^2+^:**CL1** = 1:3:0.3.

## 5. Conclusions

In this study, we prepared colloidal dispersions of coordination nanosheets, Ni_3_BHT, by adding the modulator 4,5-dihexylbenzene-1,2-dithiol (**CL1**) to BHT and nickel salt. Ni_3_BHT was modified with hexyl groups, resulting in a 10-fold higher concentration than that obtained without the modulator and a more stable dispersion than that obtained using the conventional modulator benzenedithiol. Furthermore, an analysis of various electronic and vibrational spectra using **CL1** as a label revealed that Ni_3_BHT nanoflakes partially substituted with **CL1** were formed. After consumption of BHT, the termini of the nanoflakes were modified with **CL1**. The finding that alkyl termination enhances the colloidal stability of coordination nanosheets is important for elucidating their nanostructure. The Ni_3_BHT colloids obtained in this study served as conductive inks, particularly at the **CL1**/BHT ratio of 0.3. These results suggest that Ni_3_BHT colloids have the potential as solution-processable conductive materials and provide a design strategy toward future applications in printed and flexible electronics.

## Data Availability

The original contributions of this study are included in the article/Supplementary Material. Further inquiries can be directed to the corresponding author.

## Supplementary Materials

The following supporting information can be downloaded at: https://www.mdpi.com/article/10.3390/nano16030191/s1, Figure S1: Photographs of one-month-old **Ni_3_BHT-x** and **Ni_3_BHT-BDT-x**; Figure S2: Powder X-ray diffraction patterns of **Ni_3_BHT-x**.; Figure S3: Detailed XP spectra of **Ni_3_BHT-x** in S2s region; Figure S4: The S/Ni ratio of **Ni_3_BHT-x** estimated from XPS peak area; Figure S5: S2s satellite/main peak area ratio of **Ni_3_BHT-x**; Figure S6: Raman spectra of **Ni_3_BHT-x**; Figure S7: Optical microscopy images of the interdigitated gold electrodes coated with **Ni_3_BHT-x**; Figure S8: Atomic force microscopy images of **Ni_3_BHT-x** coated on an interdigitated gold electrode; Figure S9: *I-V* curve of **Ni_3_BHT-0.3** at room temperature; Figure S10: ^1^H NMR spectra of **1**; Figure S11: ^13^C NMR spectra of **1**; Figure S12: ^1^H NMR spectra of **CL1**; Figure S13: ^13^C NMR spectra of **CL1**; Figure S14: ^1^H NMR spectra of 1-benzylthio-2-bromo-4,5-dihexylbenzene.
