# Supplementary material for: Concentrated Colloidal Dispersion of Nickelladithiolene Coordination Nanosheet Realized by an Alkylated Modulator
Source: Nanomaterials (Basel). 2026 Jan 30;16(3):191. doi: 10.3390/nano16030191 (PMC12899148; doi:10.3390/nano16030191)
Supplement: Supplementary file 1 [file nanomaterials-16-00191-s001.zip › nanomaterials-4093453-supplementary.pdf]

Supplementary Materials

# Concentrated colloidal dispersion of nickelladithiolene coordination nanosheet realized by an alkylated modulator

Naoya Fukui [1,*], Yu Endo [2], Miyu Ito [2], Kenji Takada [1], Hiroaki Maeda [1] and Hiroshi Nishihara [1,2,*]

[1] Research Institute for Science and Technology, Tokyo University of Science, 2641 Yamazaki, Noda 278-8510, Japan

[2] Department of Pure and Applied Chemistry, Faculty of Science and Technology, Tokyo University of Science, 2641 Yamazaki, Noda 278-8510, Japan

*Correspondence: n-fukui@rs.tus.ac.jp; nisihara@rs.tus.ac.jp

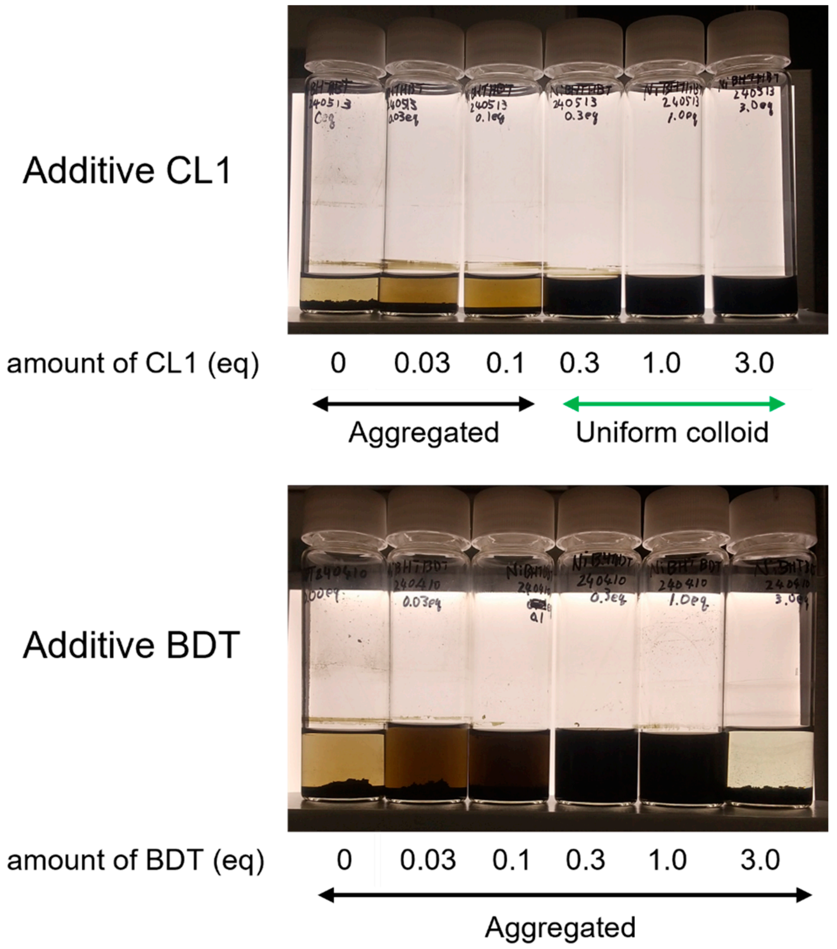


**Figure S1.** One-month-old $Ni_3BHT$ colloid with additive **CL1** (upper panel) and benzene-1,2-dithiol (BDT) (lower panel).

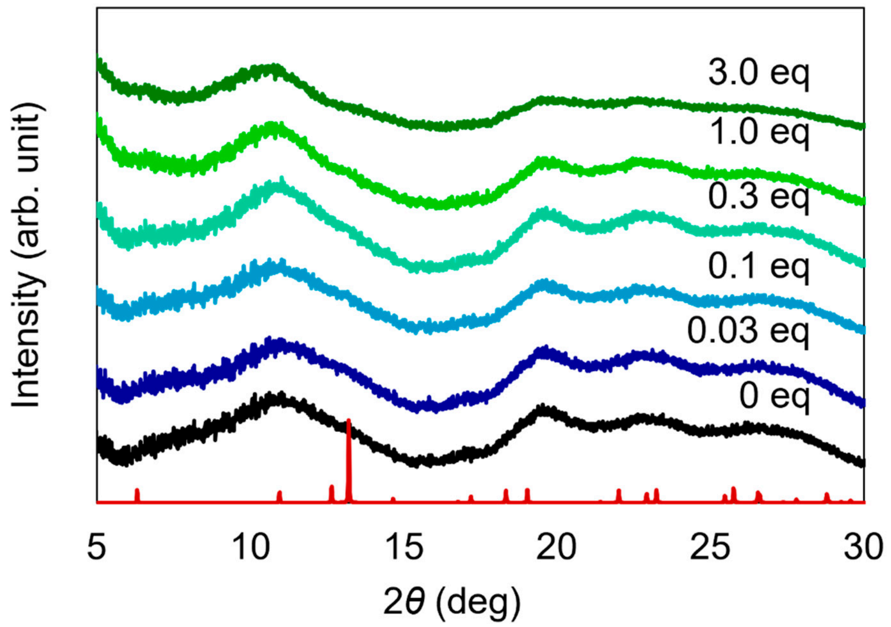


**Figure S2.** Powder X-ray diffraction patterns of **$Ni_3$BHT-x** with the simulated diffraction pattern (red line) reproduced from $Ni_3$BHT structure.

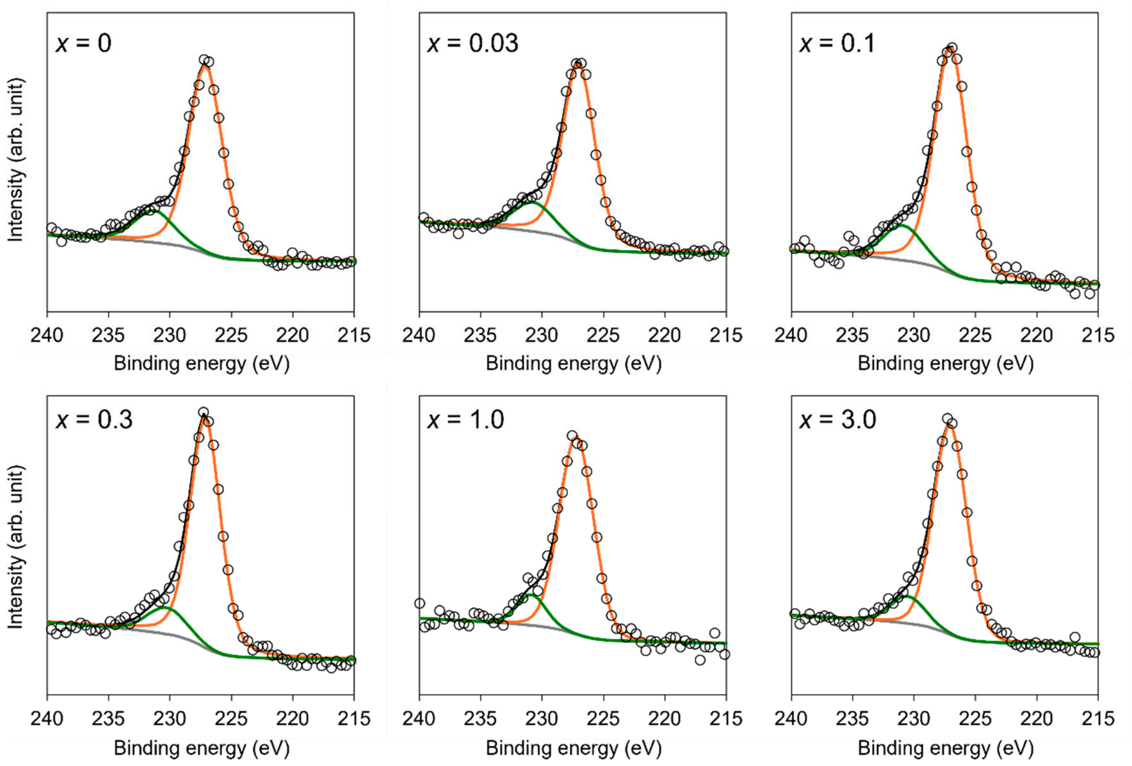


**Figure S3.** XP spectra of **Ni3BHT-x** in S2s region. The black, orange, and green lines show the fitted curves, the coordinating S peaks, and the satellite peaks, respectively.

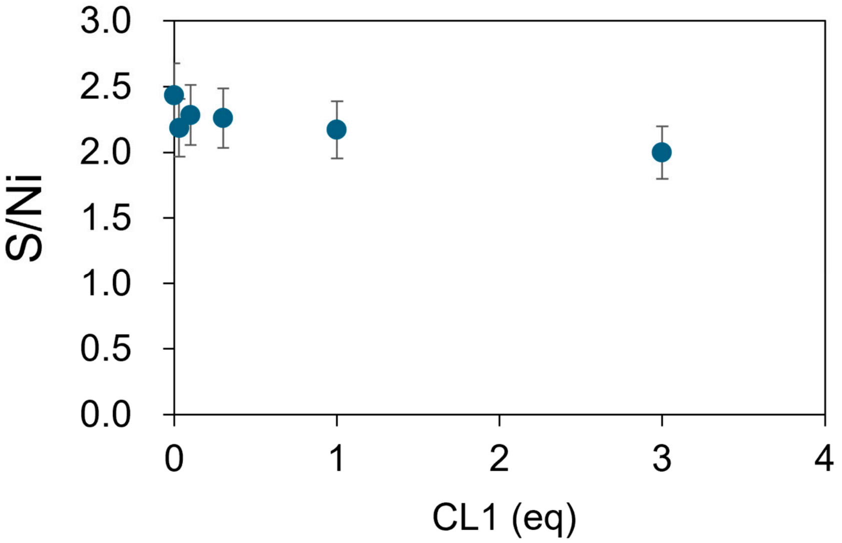


**Figure S4.** The S/Ni ratio of **$Ni_3$BHT-x** estimated from XPS peak area.

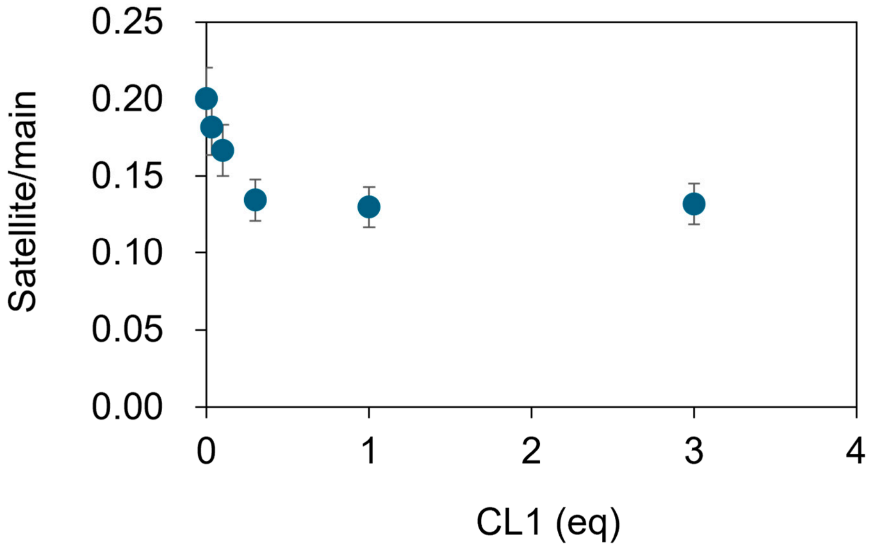


**Figure S5.** S2s satellite/main peak area ratio of **$Ni_3$BHT-x.**

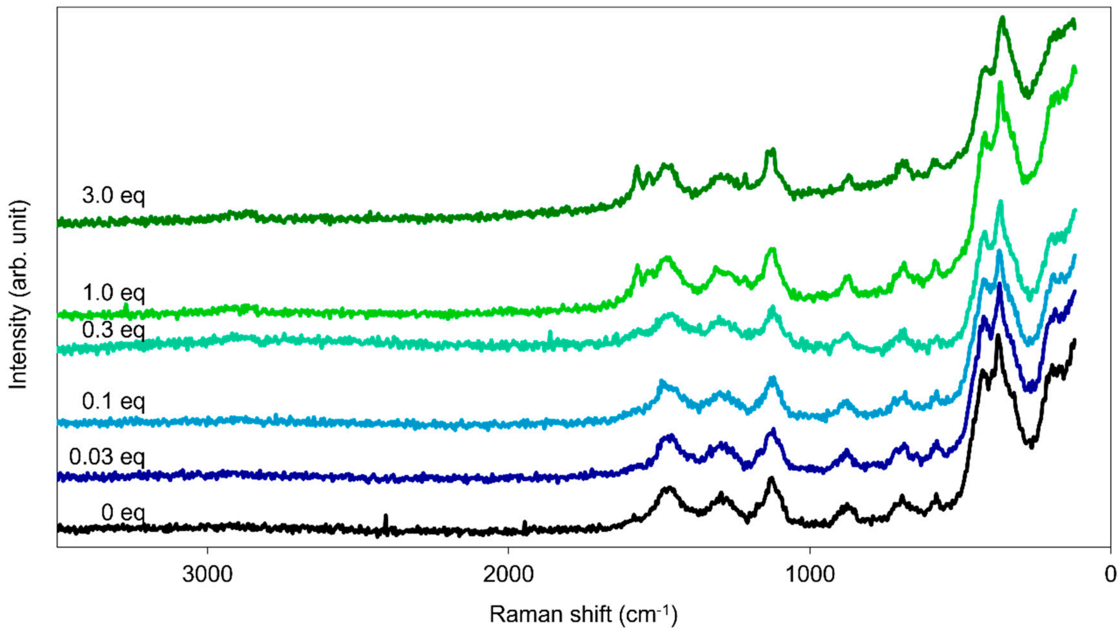


**Figure S6.** Raman spectra of **$Ni_3$BHT-x**.

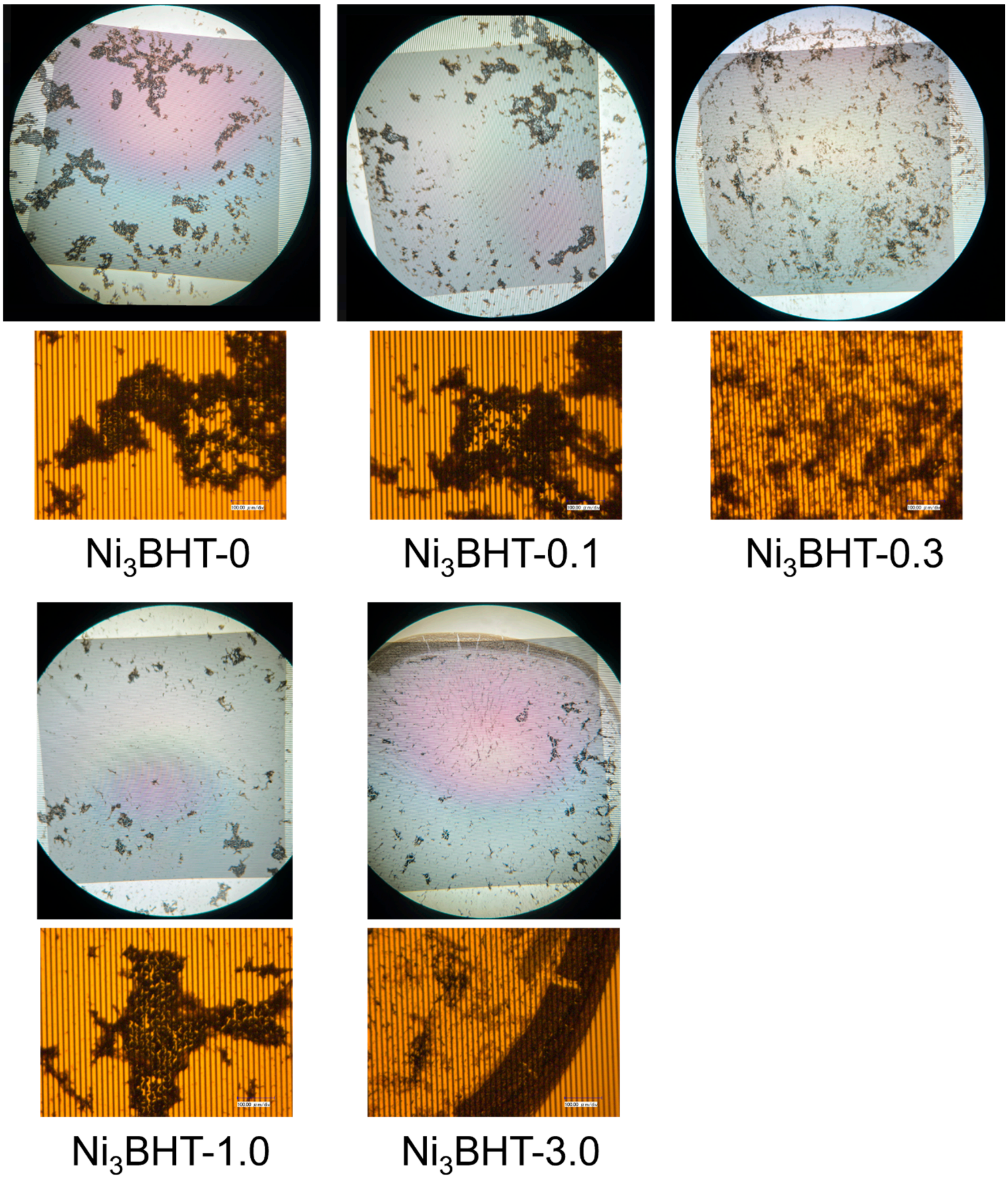


**Figure S7.** Far (upper) and close (lower) optical microscopy images of the interdigitated gold electrodes coated with **$Ni_3$BHT-x**. The diameter of the field of view is approximately 5 mm in the far images.

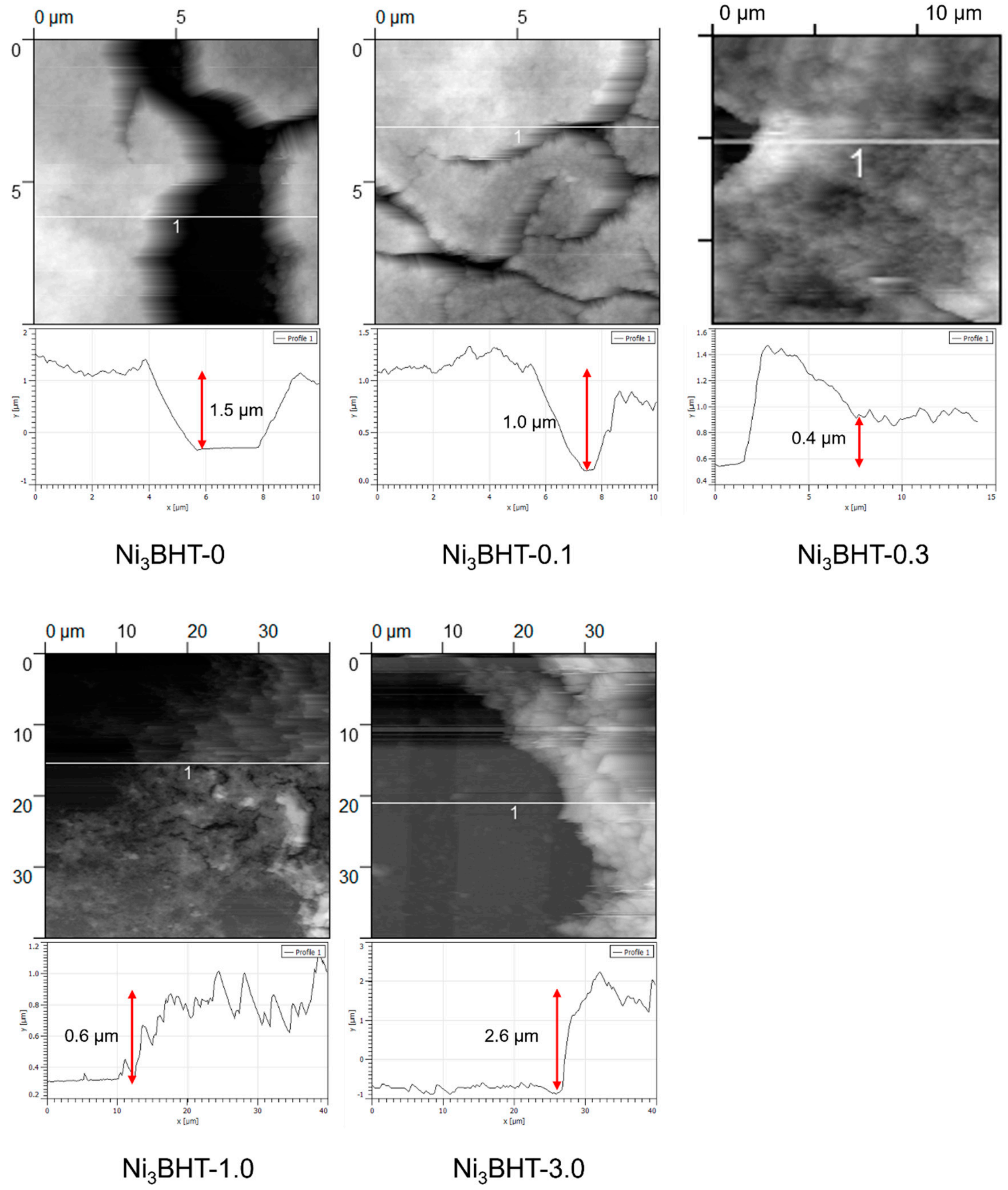


**Figure S8.** Atomic force microscope images of **$Ni_3$BHT-x** coated on an interdigitated gold electrode.

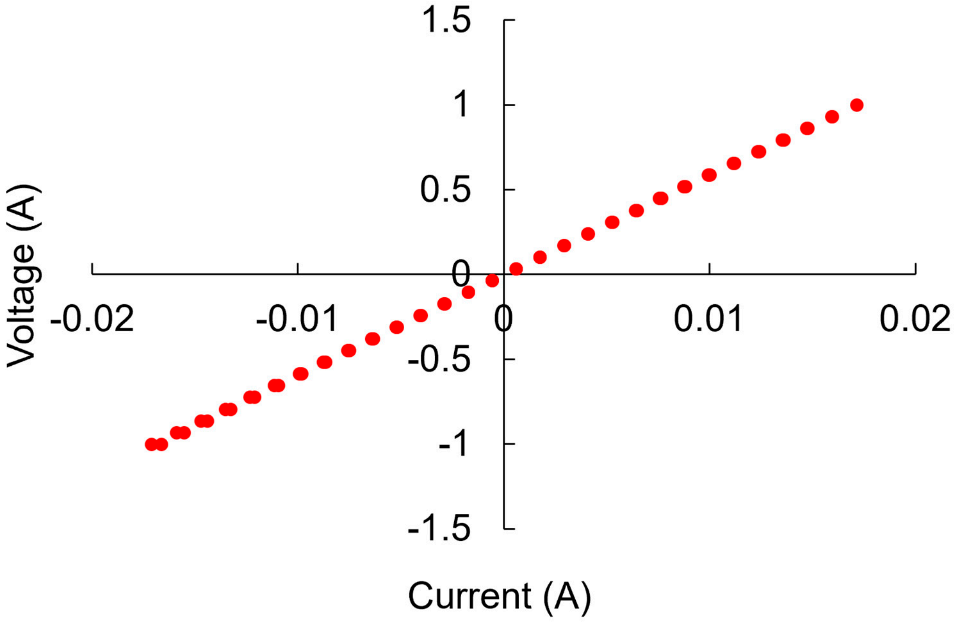


**Figure S9.** *I-V* curve of **$Ni_3$BHT-0.3** at room temperature.

**Synthesis of 1-benzylthio-2-bromo-4,5-dihexylbenzene**

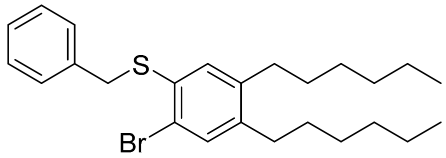


The reaction was performed in nitrogen atmosphere. Benzyl mercaptan (0.50 mL, 4.27 mmol) was added to the mixture of DMF (25 mL) and NaOH (0.192 g, 4.80 mmol), followed by the addition of **1** (500 mg, 1.24 mmol). The mixture was kept stirred at 80°C for 3 hours. After cooling, adequate amount of dichloromethane was added and repeatedly washed in a separatory funnel with water to remove DMF. The organic layer was dried over sodium sulfate, filtered, and concentrated in vacuo. The residue was purified by column chromatography on silica gel (hexane) by collecting the third band, followed by the evaporation to obtain colorless oil (159 mg, 0.355 mmol, 29%). $^1$H-NMR (400 MHz, $CDCl_3$, ppm): $\delta$ = 0.90 (t, $J$ = 6.8 Hz, 6H), 1.27-1.58 (m, 16H), 2.45 (t, $J$ = 7.9 Hz, 2H), 2.50 (t, $J$ = 7.8 Hz, 2H), 4.10 (s, 2H), 6.98 (s, H), 7.21-7.33 (m, 6H).

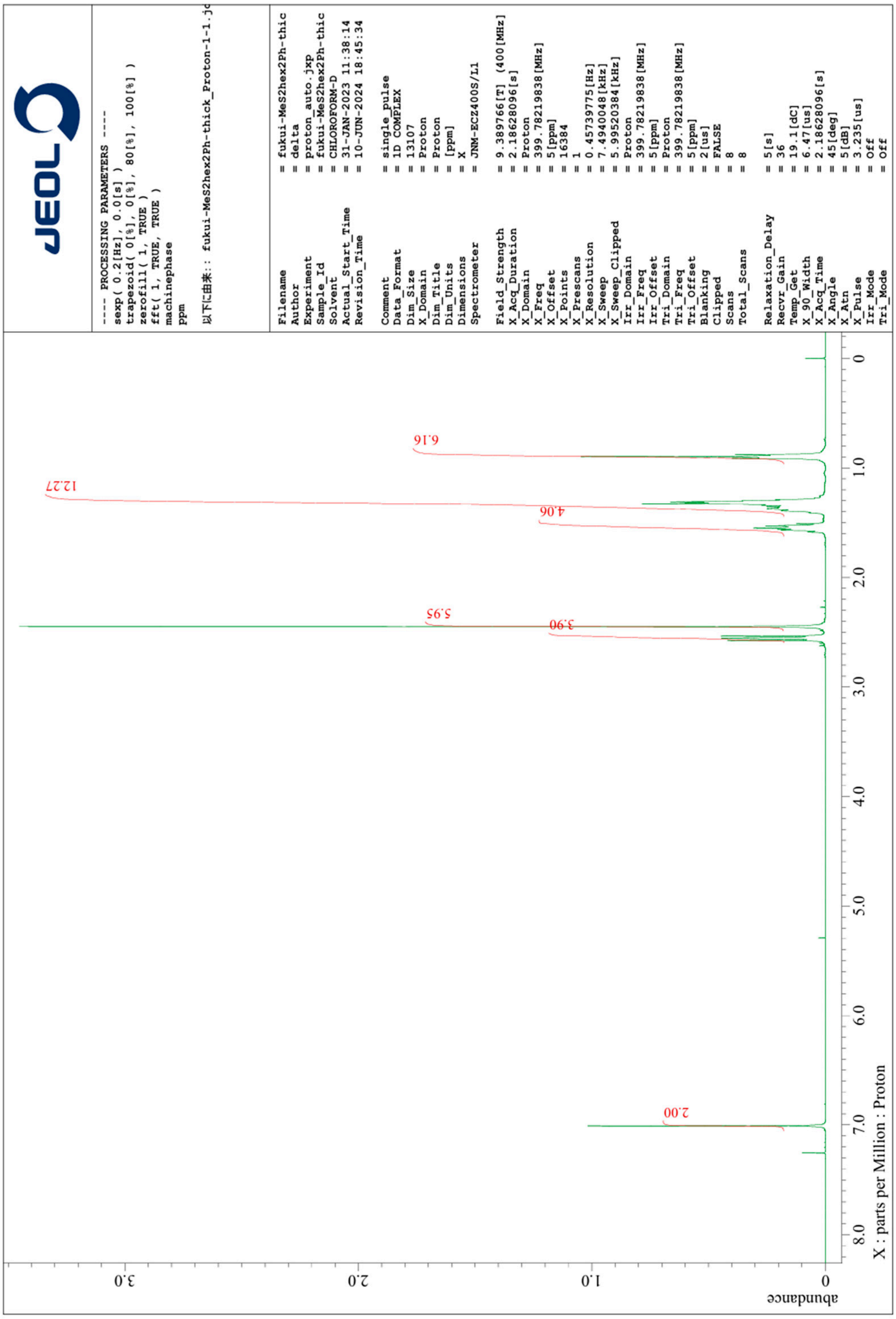


**Figure S10.** $^1$H NMR of **1** in $CDCl_3$.

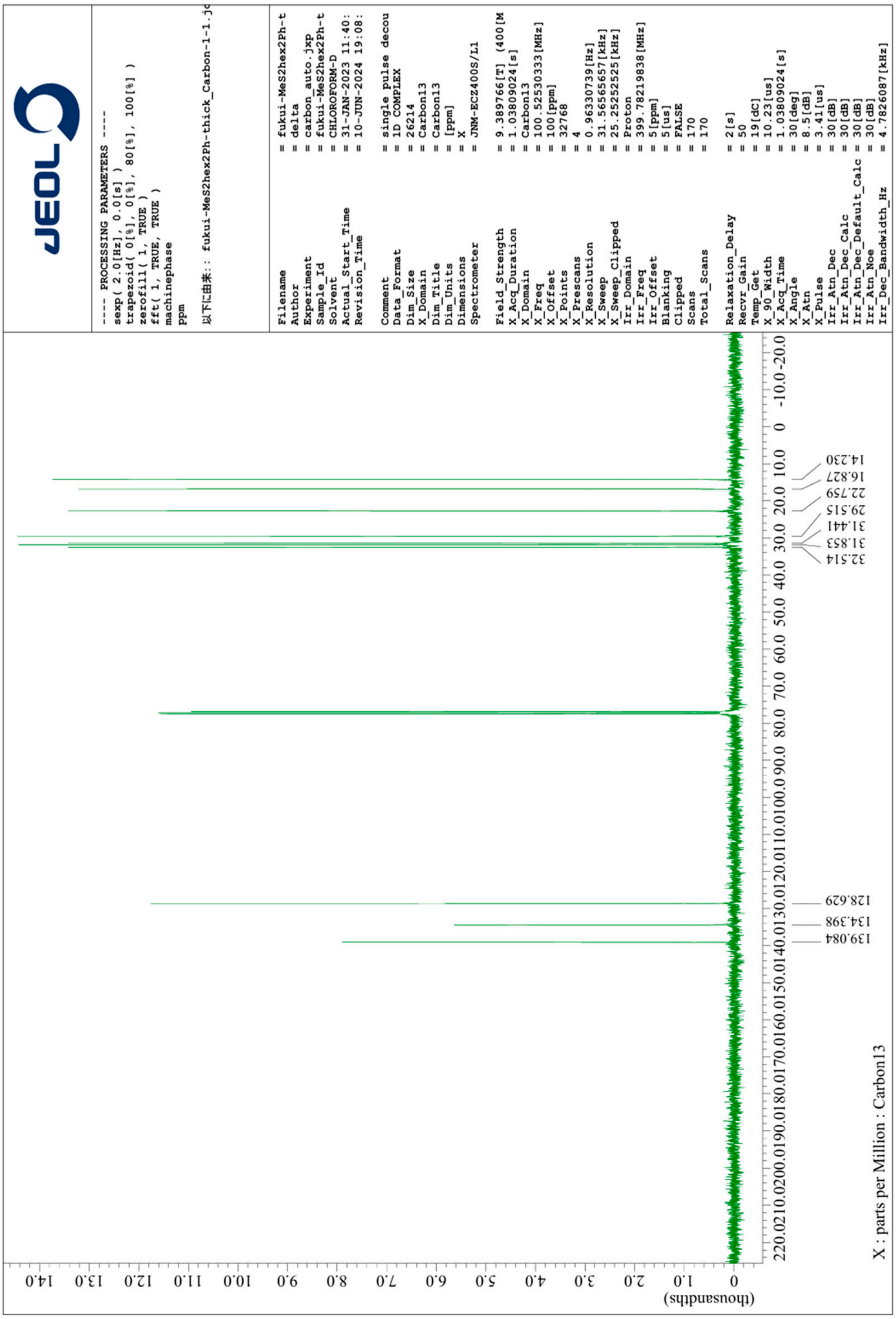


**Figure S11.** $^{13}C$ NMR of **1** in $CDCl_3$.

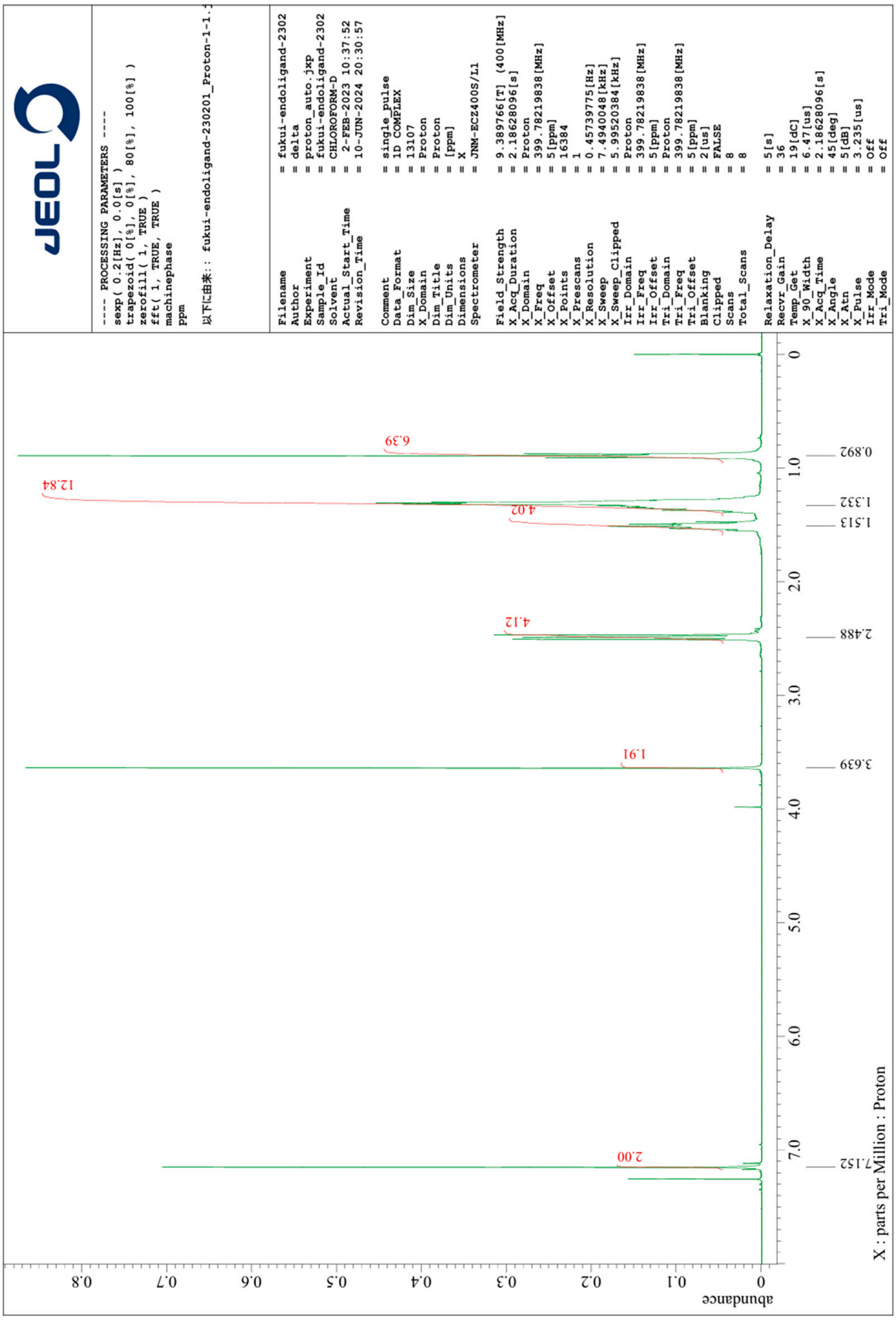


**Figure S12.** $^{1}H$ NMR of **CL1** in $CDCl_3$.

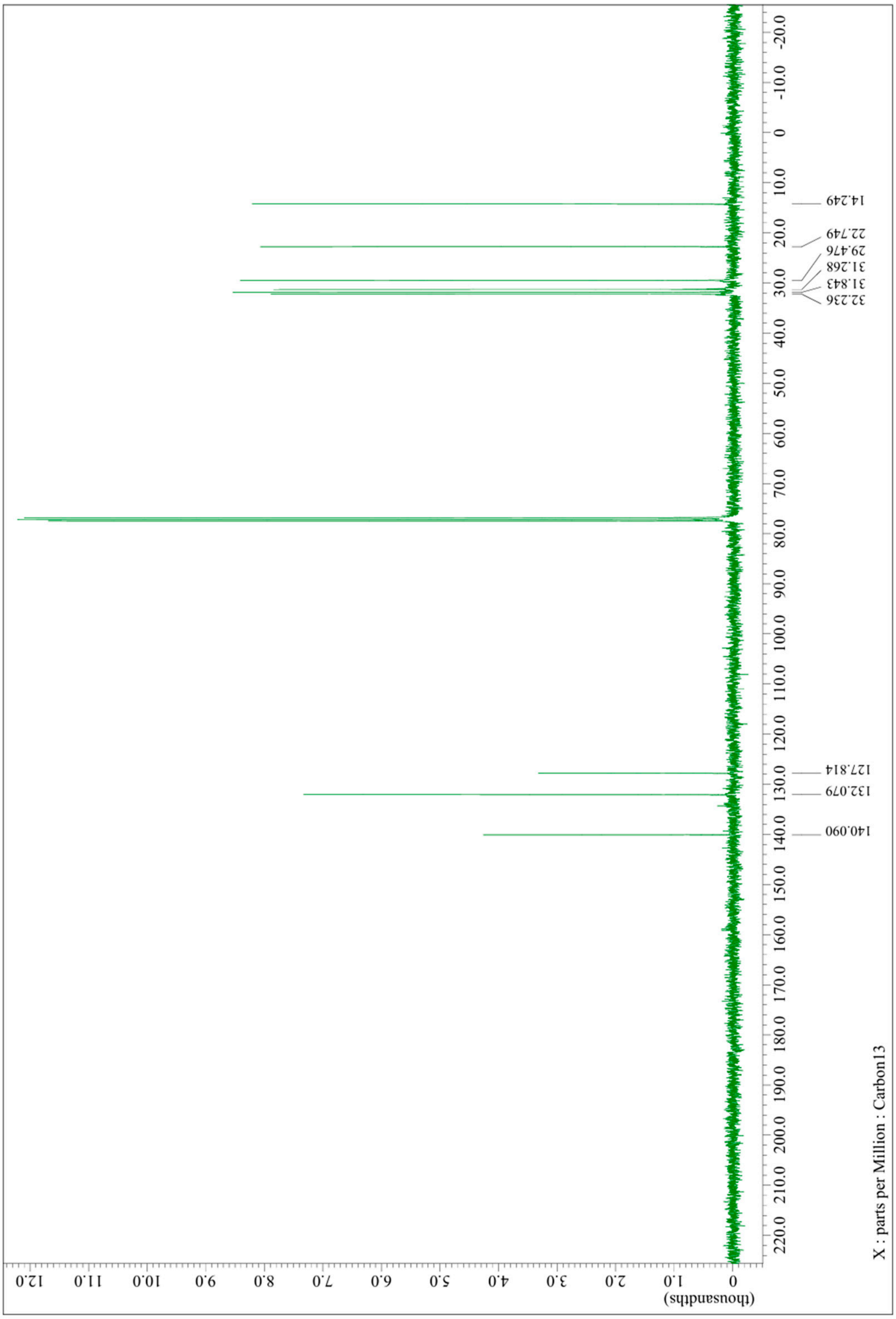


**Figure S13.** $^{13}$C NMR of **CL1** in $CDCl_3$.

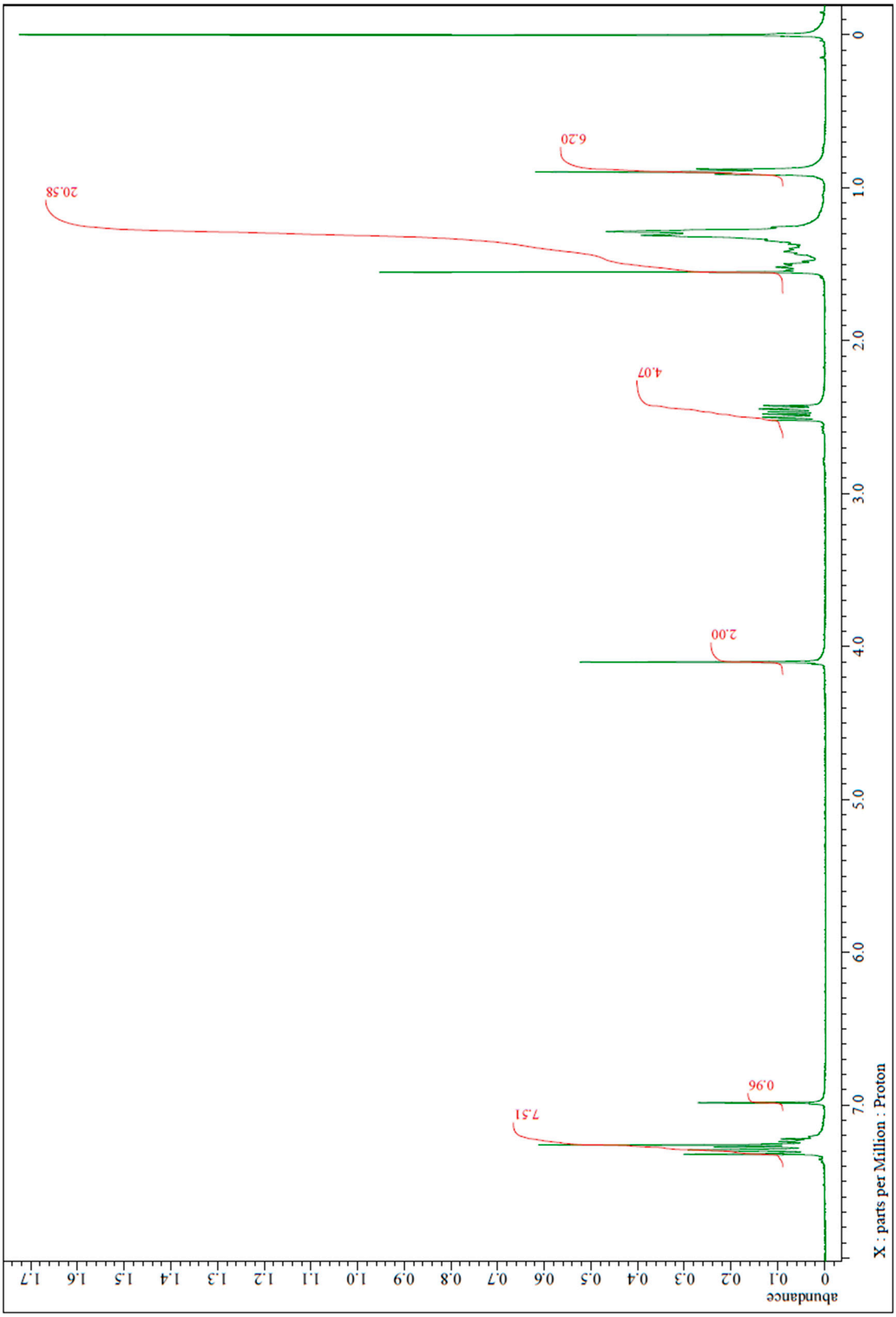


**Figure S14.** $^{1}H$ NMR of 1-benzylthio-2-bromo-4,5-dihexylbenzene in $CDCl_3$.
